# Identification of *Cysteine synthase* (*Cys*) Gene Family in Tomato (*Solanum lycopersicum*) and Functional of *SlCys5* in Cold Stress Tolerance

**DOI:** 10.3390/ijms26062801

**Published:** 2025-03-20

**Authors:** Rui Lv, Yan Gao, Xueying Yang, Xin Li, Chengyu Zhu, Fulei Mo, Kuihua Li

**Affiliations:** 1 Agricultural College, Yanbian University, Yanji 133002, China; 2 College of Horticulture and Landscape Architecture, Northeast Agricultural University, Harbin 150030, China; 3 College of Life Sciences, Northeast Agricultural University, Harbin 150030, China

**Keywords:** cold stress, *Cys*, gene family, *SlCys5*, tomato

## Abstract

Sulfur is an intermediate element in plants. It plays an important role in the growth and development of plants. Plant roots absorb sulfate from their external environment and produce cysteine under the catalysis of cysteine synthase. Cysteine is a synthetic precursor of sulfur-containing metabolites and critical molecules including glutathione (GSH), methionine, vitamins, coenzymes, and antioxidants. It also plays a central role in plant stress resistance. In this study, we identified the *Cys* family genes in tomato and analyzed the expression of *SlCys* genes under cold stress. A bioinformatics analysis showed that the *SlCys* gene promoters were rich in *cis*-acting elements related to stress response. Transcriptome data analysis and qRT-PCR (real-time fluorescent quantitative polymerase chain reaction) experiments showed that *SlCys5* may be the key gene in the *Cys* gene family for cold tolerance in tomato. After cold stress treatment, the *SlCys5*-silenced tomato plants were more sensitive to cold stress, and wilting was more severe than in control plants. Thus, *SlCys5* is a positive regulator of cold tolerance in tomato. In this study, we elucidated the evolutionary pattern and functional differentiation of the *Cys* gene family in tomato, deepening our understanding of the regulatory mechanism of cold stress tolerance in plants.

## 1. Introduction

Sulfur is an intermediate element in plants. It plays an important role in the growth and development of plants [1]. Plants obtain sulfur from soil in the form of sulfate taken up by the roots. Cysteine, the first sulfur compound, is then formed under the catalysis of cysteine synthase [2]. In the final step of cysteine synthesis, cysteine synthetase catalyzes the combination of H_2_S and O-acetylserine to generate cysteine [3,4]. Cysteine is a precursor not only for the synthesis of sulfur-containing metabolites (glutathione, methionine, etc.), but also for many important molecules in plants (vitamins, coenzymes, antioxidants, etc.) [5]. Cysteine is at the center of plant metabolism and plays an important role in plant life processes, especially stress resistance.

In plants, cysteine synthase can regulate metal stress resistance and harmful oxide clearance by regulating cysteine synthesis to affect GSH content [6,7]. In one study, the cytosolic *CysA* gene and the chloroplast *CysB* gene from spinach (*Spinacia oleracea*) were each transformed into tobacco (*Nicotiana tabacum*). When the two transgenic plants were crossed, it was found that GSH content, cysteine content, and tolerance to heavy metals (Se, Cd, Ni, etc.) were all significantly higher in F1 hybrid plants than in control plants and parents [8]. In another study, the overexpression of *Atcys-3A* in Arabidopsis (*Arabidopsis thaliana*) was found to significantly increase the tolerance of transgenic plants to CdCl_2_, compared with wild type [9]. Overexpression of the soybean (*Glycine max*) *Cys* family gene in tobacco improves the tolerance of tobacco to oxygen stress [10]. The Cys family proteins also have other functions, in addition to catalyzing the combination of H_2_S and O-acetylserine to cysteine. The Arabidopsis Cys family protein DES1 functions as a cysteine desulfurase to catalyze the desulfurization of cysteine, releasing hydrogen sulfide and enhancing the adaptability of plants to their environment [11]. In addition, the Cys family protein SCS in thylakoids can function as S-thiocystine synthetase and participate in photosynthesis [12]. The CYS-C1 protein in Arabidopsis can function as β-cyanopropionate synthase and CYS-C1 to participate in the detoxification cycle of cyanide, converting cyanide and cysteine to β-cyanoalanine and hydrogen sulfide, respectively, and thereby mitigate cyanide damage to plants [13].

All members of the Cys family in plants contain conserved pyridine 5′-phosphate binding sites, which were first reported in Arabidopsis [13]. Although the *Cys* family has been reported in sorghum (*Sorghum vulgare*), foxtail millet, alfalfa (*Medicago sativa* L.), and so on [14,15,16], the *Cys* gene family in the complete genome of tomato has not yet been identified. As a common horticultural crop, tomato is not only widely cultivated, but is also often studied scientifically as a model plant. The mining of its stress resistance genes is of great significance. In this study, all six of the *Cys* family genes in tomato were identified. The gene structure, location of genes, *cis*-acting elements in the promoter, collinearity among *SlCys* family genes, and motifs in the phylogenetic tree of Cys proteins were all analyzed. The expression pattern of *SlCys* genes in tomato under cold stress was also analyzed, and the function of *SlCys5* in cold stress tolerance in tomato was revealed through silencing the *SlCys5* gene. In this study, we elucidated the evolution and functional differentiation of tomato *Cys* family genes, providing a theoretical basis for further understanding of the regulatory mechanism of cold tolerance in plants.

## 2. Results

### 2.1. Identification of Cys Family Members in Tomato

The Cys protein in Arabidopsis (protein number: AT3G04940.1) was downloaded from TAIR, and the tomato total protein file was downloaded from the Ensembl Plants website. Hidden Markov models (HMMs) of the Cys protein in other plants were downloaded according to InterPro number (TIGR01136). Arabidopsis Cys protein was submitted to the tomato total protein file using BLAST to search for similar sequences, and HMM search was performed using TBtools (v2.138). The results of BLAST and HMM searches were intercrossed, and six *Cys* genes were ultimately identified in tomato. These *Cys* genes were named *SlCys1*–*SlCys6*, based on their location on the tomato chromosome. The *SlCys* family genes encode proteins with amino acid lengths between 323 (SlCys2) and 421 (SlCys3), and molecular weights between 34.24255 kDa (SlCys5) and 45.0241 kDa (SlCys3). Values for the pI (isoelectric point) of SlCys family proteins were between 5.28 (SlCys2) and 8.39 (SlCys6) (Table S1).

### 2.2. Localization Analysis of Tomato SlCys Genes on Chromosomes

**Figure 1:**
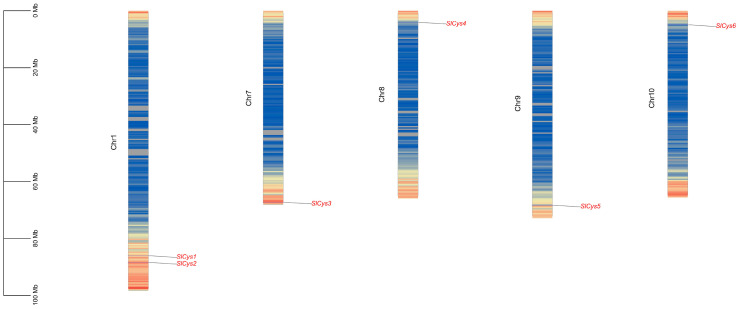
Localization analysis of *SlCys* genes on tomato chromosomes. Scale indicates the length of the chromosome. Chromosome color from blue to red indicates gene density (blue: less dense; red: more dense).

The localization of *SlCys* genes on chromosomes and the gene density of tomato chromosomes were extracted according to the tomato genome file and the genome annotation file, respectively. The localization of *SlCys* family genes on tomato chromosomes was visualized using TBtools (v2.138). The results of the chromosome localization analysis showed that all six *SlCys* genes were unevenly distributed on five of the twelve chromosomes (chromosomes 1, 7, 8, 9, and 10), and *SlCys* family genes were more likely to be distributed on both ends of the chromosomes. Chromosome 1 contains two *SlCys* genes, while other chromosomes containing the *SlCys* genes contain only one *SlCys* gene (Figure 1).

### 2.3. Analysis of Protein-Conserved Motifs and Gene Structures of SlCys Family Members in Tomato

**Figure 2:**
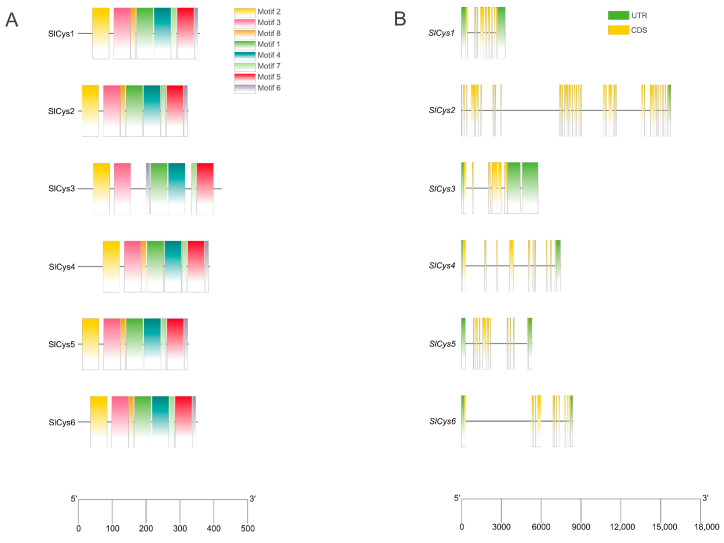
(**A**) Conserved motif analysis of SlCys family proteins, with scale indicating amino acid length. (**B**) Structural analysis of *SlCys* genes. The scale indicates gene length and black lines indicate introns.

A total of eight types of motif were identified in tomato SlCys family proteins, and motifs 1, 2, 3, 4, 5, 7, and 8 were found in all SlCys proteins. The SlCys3 protein contained the fewest motifs (seven motifs) among the SlCys family members. The other SlCys proteins all contained eight motifs. Motif 6 was not present in SlCys3 (Figure 2A). The gene structures of *SlCys* genes were analyzed according to the tomato genome annotation file. The results showed that all *SlCys* genes contained introns; among these genes, *SlCys1* had the shortest intron length and *SlCys2* had the longest intron length (Figure 2B).

### 2.4. Phylogenetic Analysis of Cys Proteins and Collinearity Analysis of Cys Family Genes

**Figure 3:**
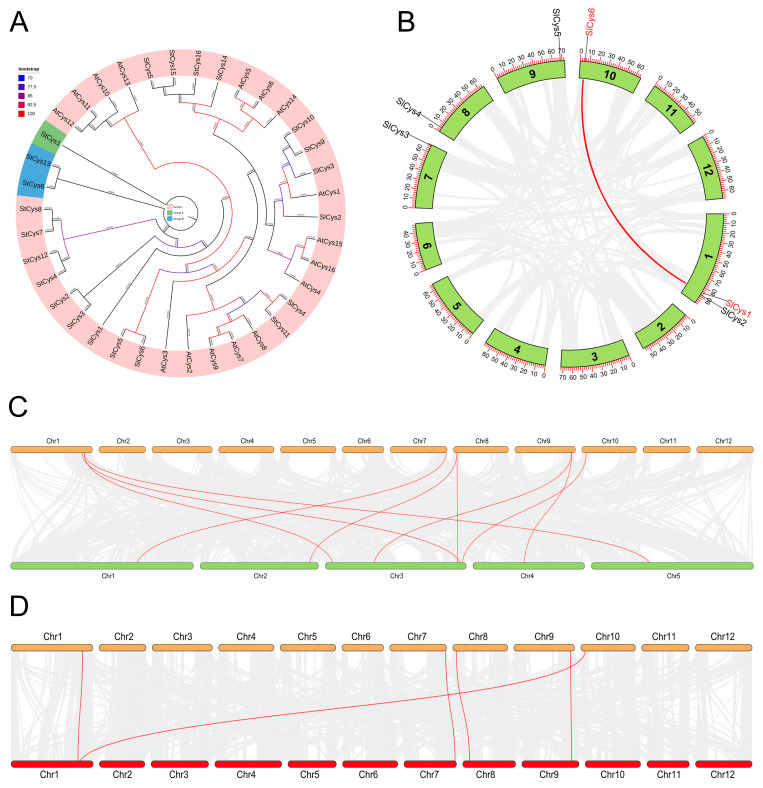
(**A**) Phylogenetic tree of tomato, Arabidopsis, and potato Cys proteins. (**B**) Collinearity among *SlCys* family genes in tomato. The scale indicates chromosome length, and the gray lines indicate collinearity among tomato non-*SlCys* family genes. (**C**) Synteny between tomato *Cys* genes and Arabidopsis *Cys* genes. Tomato chromosomes are shown in orange and Arabidopsis chromosomes in green. Gray lines represent synteny between non-*Cys* genes, and red lines represent synteny between *Cys* genes. (**D**) Synteny between tomato *Cys* genes and potato *Cys* genes. Tomato chromosomes are shown in orange and potato chromosomes in red. Gray lines represent synteny between non-*Cys* genes, and red lines represent synteny between *Cys* genes.

Cys proteins in Arabidopsis and potato (*Solanum tuberosum*) were identified using BLAST and HMM search methods. A total of 16 Cys proteins were identified in the full genome scope of Arabidopsis; these were named AtCys1 to AtCys16. A total of 16 Cys proteins were identified in the full genome scope of potato; these were named StCys1 to StCys16. Phylogenetic analyses of tomato, Arabidopsis, and potato Cys proteins were performed based on the sequence of Cys proteins. The phylogenetic tree was then visualized. All of the Cys proteins (tomato, Arabidopsis, and potato) in the phylogenetic tree were divided into three groups (Groups I, II, and III) and several subgroups according to their genetic relationship. Most tomato Cys proteins, Arabidopsis Cys proteins, and potato Cys proteins were grouped into the same group (Group I). Group III contained two potato Cys proteins (StCys6 and StCys13). Group II contained only one potato Cys protein (Figure 3A). Notably, tomato Cys proteins were more likely than Arabidopsis proteins to be grouped into the same subfamily as potato Cys proteins.

To understand the duplication events in the tomato *SlCys* family genes, a collinearity analysis of six *SlCys* genes was carried out. This revealed collinearity between *SlCys1* and *SlCys6* in tomato (Figure 3B). The synteny between tomato *Cys* and other plant *Cys* genes was also analyzed to understand the evolutionary mechanism of *Cys* genes. The results showed that all tomato *Cys* genes were in synteny with the Arabidopsis *Cys* genes (*SlCys1*/*SlCys6*-*AT3G61440.1*; *SlCys2*-*AT3G04940.1*/*AT5G28020.1*; *SlCys3*-*AT1G55880.1*; *SlCys4*-*AT2G43750.2*/*AT3G59760.1*; *SlCys5*-*AT3G22460.1*/*AT4G14880.2*) (Figure 3C). Five *SlCys* genes were in synteny with potato *Cys* genes, such as *SlCys1*/*SlCys6* with *PGSC0003DMT400000156*, *SlCys3* with *PGSC0003DMT400057393*, and *SlCys4* with *PGSC0003DMT400059624* (Figure 3D).

### 2.5. Analysis of Cis-Acting Elements in Tomato SlCys Promoter

**Figure 4:**
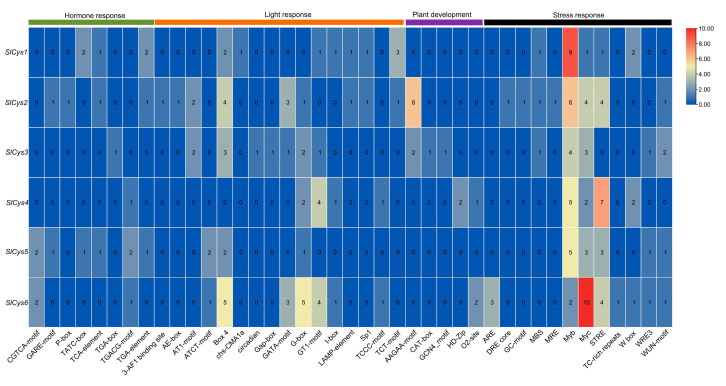
Heat map of *cis*-acting elements in the promoters of tomato *SlCys* family genes.

To understand the *cis*-acting elements contained in the promoters of *SlCys* family genes, the sequences (2000 bp before the initial codon) of the promoter regions of all *SlCys* genes were extracted and used to identify *cis*-acting elements. A total of 41 *cis*-acting elements were identified in the ten *SlCys* gene promoters. These were classified into four types: hormone, light, plant development, and stress response (Figure 4). The results showed that the *SlCys* gene promoters contained more light-response- and stress-response-type *cis*-acting elements, such as AE-box, ATCT-motif, GT1-motif, STRE, ARE, and MYC elements. In addition, the *SlCys* gene promoters also contained a small number of element types related to plant development and hormone response, such as GARE-motif, TCA-element, AAGAA-motif, and HD-Zip elements, indicating the functional diversity of tomato *Cys* family genes.

### 2.6. Transcriptome Data and qRT-PCR Analysis of SlCys Family Genes Under Cold Stress

**Figure 5:**
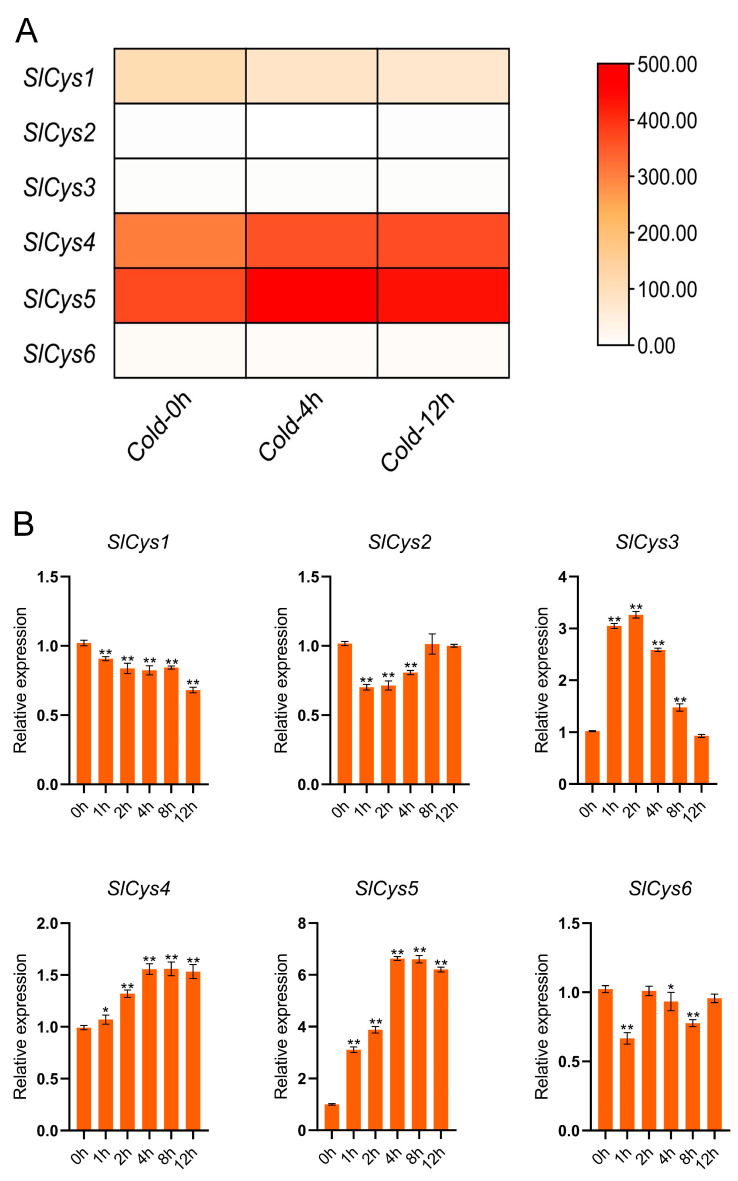
(**A**) Heatmap of transcriptome data of *SlCys* genes under cold stress. (**B**) Expression patterns of *SlCys* family genes under cold stress based on qRT-PCR experiments. Error bars indicate the standard error of the mean (*n* = 3). The significance test was performed using the one-way ANOVA method, with the significance mark (*) indicating *p* < 0.05 and (**) indicating *p* < 0.001.

To understand the expression pattern of *SlCys* family genes under cold stress, transcriptome data of tomato plants under cold treatment were analyzed. The FPKM values of *SlCys* family genes were visualized. The results showed that all *SlCys* family genes were expressed in the transcriptome data, but *SlCys2*, *SlCys3,* and *SlCys6* had lower FPKM values. Under cold stress, the expression of *SlCys4* and *SlCys5* was upregulated and the expression of *SlCys1* was downregulated (Figure 5A).

To verify the accuracy of the transcriptome analysis, and to analyze the expression of *SlCys* genes under cold stress in more depth, we analyzed the expression patterns of *SlCys* family genes under cold stress using qRT-PCR experiments. The results of these experiments showed that the relative expression levels of *SlCys3*, *SlCys4,* and *SlCys5* were upregulated under cold stress. The relative expression of *SlCys1* and *SlCys2* was downregulated. The expression pattern of *SlCys6* was irregular (Figure 5B). Among the upregulated *SlCys* family genes, the relative expression of *SlCys4* and *SlCys5* did not increase after 4 h of cold stress. The expression of *SlCys3* first increased and then decreased, but overall expression was always upregulated. In addition, the relative expression level of *SlCys5* was significantly higher than that of other *SlCys* genes, suggesting that *SlCys5* not only responds to cold stress, but also may play a role in tomato cold stress resistance.

### 2.7. Silencing of the SlCys5 Gene Reduced Cold Tolerance in Tomato

**Figure 6:**
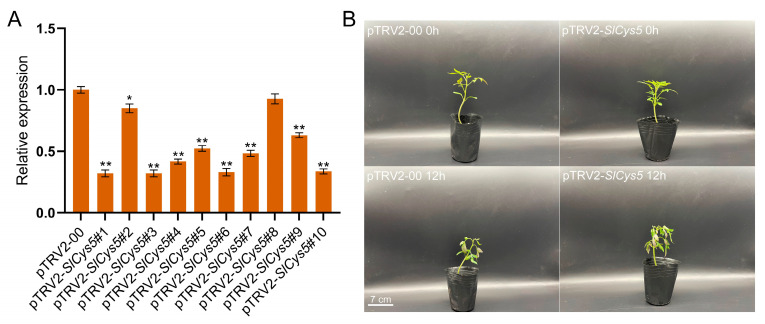
(**A**) Silencing efficiency assay of the *SlCys5* gene in tomato. Error bars indicate the standard error of the mean (*n* = 3). The significance test was performed using the one-way ANOVA method, with the significance mark (*) indicating *p* < 0.05 and (**) indicating *p* < 0.001. (**B**) Phenotypes of *SlCys5*-silenced plants and control plants under cold stress.

The expression of *SlCys5* in tomato *Cys* genes increased most significantly under cold stress. We therefore hypothesized that *SlCys5* was a potential gene for maintaining cold tolerance in tomato and verified the function of the *SlCys5* gene in tomato under cold stress using the virus-induced gene-silencing (VIGS) method. We found no significant difference in morphology between pTRV1/pTRV2-00-transfected tomatoes (control plants) and pTRV1/pTRV2-*SlCys5*-transfected tomatoes (*SlCys5*-silenced plants) at 20 days after transfection. We then measured the relative expression of the *SlCys5* gene in control plants and *SlCys5*-silenced plants using qRT-PCR. The results showed that expression of the *SlCys5* gene in most *SlCys5*-silenced plants (pTRV2-*SlCys5*#1, 3, 4, 5, 6, 7, 9, 10) was significantly lower than in the control plants (Figure 6A), indicating that the *SlCys5* gene was successfully silenced in tomato. Plants with higher silencing efficiency (pTRV2-*SlCys5*#1, 3, 4, 6, 7, 10) were selected for subsequent experiments.

**Figure 7:**
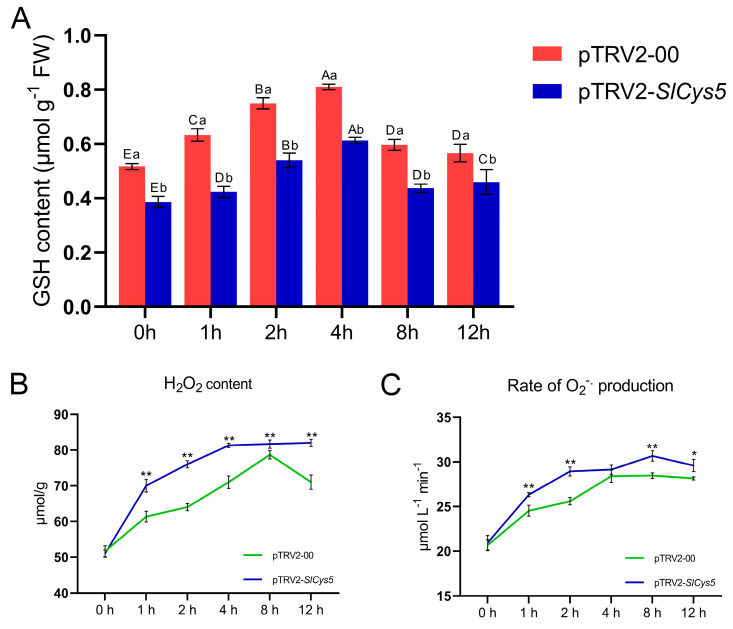
(**A**) GSH content in *Slcys5*-silenced plants and control plants under cold treatment. Error bars indicate the standard error of the mean (*n* = 3). Significance was analyzed at the 0.05 significance level using the two-way ANOVA method. Capital letters indicate significant differences between different time courses of the same experimental plant material, and lowercase letters indicate significant differences between different experimental plant materials at the same time. (**B**) H_2_O_2_ content in *Slcys5*-silenced plants and control plants under cold treatment. (**C**) Rate of O_2_^−•^ production in *Slcys5*-silenced plants and control plants under cold treatment. Error bars indicate the standard error of the mean (*n* = 3). The significance test was performed using the one-way ANOVA method, with the significance mark (*) indicating *p* < 0.05 and (**) indicating *p* < 0.001.

*SlCys5*-silenced plants and control plants were subjected to cold treatment at a temperature of 0 °C, and the phenotypes of experimental plants were observed after 12 h of cold treatment. The results showed that both *SlCys5*-silenced plants and control plants showed varying degrees of wilting, with *SlCys5*-silenced plants showing more severe wilting and poorer cold tolerance (Figure 6B). GSH content in *SlCys5*-silenced plants and control plants increased first and then decreased under cold stress. At 0, 1, 2, 4, 8, and 12 h of cold treatment, GSH content in *SlCys5*-silenced plants was significantly lower than that of control plants (Figure 7A). Notably, GSH content in *SlCys5*-silenced plants and control plants increased first and then decreased under cold stress. At 0, 1, 2, 4, 8, and 12 h of cold treatment, GSH content in *SlCys5*-silenced plants was significantly lower than that of control plants (Figure 7A). The content of H_2_O_2_ in control plants first increased and then decreased, while the content of H_2_O_2_ in *SlCys5*-silenced plants continued to increase and remained significantly higher than in control plants (Figure 7B); moreover, the rate of O_2_^−•^ production in *SlCys5*-silenced plants was also significantly higher than in control plants (Figure 7C).

## 3. Discussion

Tomato is widely grown and sold as a vegetable around the world. Due to the different growing environments in planting areas, tomato plants often face a variety of environmental stresses (drought, flood, saline–alkali, high temperature, low temperature, etc.) [17]. Under cold stress, photosynthesis and respiration are reduced in plants [18,19,20], causing their metabolism to be disordered; this is accompanied by severe oxidative damage [21,22]. Extreme low temperatures can also lead directly to plant death. Plants use various mechanisms to regulate cold tolerance; among these, the regulation of ROS homeostasis is crucial. The ASA-GSH cycle is one of the important pathways for ROS scavenging in plants [23]. Cysteine synthetase, as a precursor of GSH synthesis [24], plays a positive role in antioxidant and stress resistance in plants [14,25,26]. In this study, a total of six *Cys* family genes were identified in the full genome scope of tomato (Table S1). All *SlCys* genes were unevenly distributed at both ends of the different chromosome, indicating that *SlCys* family genes may all have high expression activity, as is frequently observed in other gene family studies [27,28,29]. We analyzed the gene structures and protein conservation motifs of *SlCys* family members (Figure 2), as well as the phylogenies of tomato, Arabidopsis and potato Cys proteins (Figure 3A). Our results showed that, compared with Arabidopsis, tomato Cys proteins were more likely to be grouped into the same subfamily as potato Cys proteins in the phylogenetic tree, which may be due to the closer relationship between tomato and potato. We also found collinearity among the tomato *SlCys* genes (Figure 3B) and synteny between *SlCys* genes and *Cys* genes of other species (Figure 3C,D), indicating that *Cys* family genes in plants may have the same ancestor, which recurred in their evolution.

The promoter regions of *SlCys* genes were analyzed, and the *cis*-acting elements contained in their promoters were statistically analyzed. In addition to the *cis*-acting elements necessary for plant growth and development (such as light-responsive and hormone-responsive elements), the *SlCys* promoters were also rich in abiotic stress-responsive *cis*-acting elements (Figure 4). This may indicate the diversification of *SlCys* gene functions as well as their unique functions in abiotic stress resistance. Plants under stress adjust their expression of genes to increase their likelihood of survival [30]. In order to understand the expression pattern of *SlCys* family genes in tomato under cold stress, we used qRT-PCR to analyze the expression of *SlCys* genes under cold stress based on the transcriptome data of tomato under cold stress, applying a more intensive time interval. The results showed that the expression levels of all *SlCys* family genes in tomato changed significantly under cold stress, and *SlCys5* was significantly upregulated (Figure 5). This indicated that *SlCys* family genes may play a role in cold stress resistance, and that *SlCys5* may be the key gene regulating tomato cold stress resistance in this family. We successfully silenced the *SlCys5* gene in tomato using the VIGS method (Figure 6A), and the wilting degree in *SlCys5*-silenced plants was found to be significantly higher than in control plants under cold stress (Figure 6B), indicating that *SlCys5* is a positive regulator of cold tolerance in tomato. The GSH content of *SlCys5*-silenced plants and control plants first increased and then increased under cold stress, but GSH content in *SlCys5*-silenced plants was significantly lower than in control plants at the same time points (Figure 7A). Cysteine synthetase is the synthetic precursor of GSH [5]; therefore, the decrease in GSH content in *SlCys5*-silenced plants may have been due to impaired GSH synthesis after *SlCys5* silencing. Previous studies have shown that plants can experience oxidative stress under stress [31], and that an excess of reactive oxygen species (such as H_2_O_2_ and O_2_^−•^) can cause oxidative damage to cells [32]. Under cold stress, the content of H_2_O_2_ and the rate of O_2_^−•^ production in *SlCys5*-silenced plants were significantly higher than in control plants (Figure 7B,C); these findings confirmed the positive regulatory role of *SlCys5* in cold tolerance in tomato. The ASA-GSH cycle plays a positive role in ROS scavenging [23]. Impaired GSH synthesis in *SlCys5*-silenced plants leads to a decreased ROS scavenging capacity, which may be the reason for their decreased cold tolerance. In summary, *SlCys5* may positively regulate cold tolerance in tomato by increasing intracellular ROS clearance through the regulation of GSH synthesis.

## 4. Materials and Methods

### 4.1. Plant Materials and Treatments

Tomato seeds (Ailsa Craig) provided by the Tomato Genetics Resource Center were used in this study. All seeds were seeded into soil blocks and grown in plant incubators. Plant incubator parameters were as follows: light: 16 h, with a light intensity of 160 μM, photons m^−2^s^−1^, and temperature of 25.5 °C; dark: 8 h, with a temperature of 19.5 °C. Tomato seedlings were treated with cold stress at 0 °C when they were 20 days old. Tomato leaves at 0, 1, 2, 4, 8, and 12 h after cold stress were used for subsequent experiments.

### 4.2. Identification and Sequence Analysis of Tomato Cys Family Members

The tomato genome file (version 3.0) and genome annotation file (version 3.0) were downloaded from Ensembl Plants (https://plants.ensembl.org/index.html, accessed on 2 November 2024). The Arabidopsis Cys protein sequence (AT3G04940.1) was downloaded from TAIR (https://www.arabidopsis.org/, accessed on 2 November 2024). All of the CDS in tomato were extracted from the tomato genome file and translated into protein sequences. The Arabidopsis Cys protein sequence was submitted to the tomato protein sequence for BLAST to search for similar sequences. The HMM was selected based on existing studies of Cys protein domains and the identification of *Cys* family genes in other crops [13,14,15,16]. HMM files of the Cys protein were downloaded according to their InterPro number (TIGR01136) from InterPro (https://www.ebi.ac.uk/interpro/, accessed on 2 November 2024). HMM files of the Cys proteins were submitted to tomato protein sequences for HMM search using TBtools (v2.138) [33]. BLAST and HMM search results were intercrossed, and domains were confirmed. Finally, the Cys family members in tomato were identified. The sequences of Cys protein in tomato were submitted to the Expasy website (https://web.expasy.org/protparam/, accessed on 2 November 2024) for an analysis of their physical and chemical properties.

### 4.3. Chromosomal Localization Analysis of Tomato Cys Family Genes

The location of the *SlCys* family genes on tomato chromosomes and the number, length, and density of the tomato chromosomes were extracted from the tomato genome annotation file. The chromosomal localization of *SlCys* family genes in tomato were visualized using TBtools (v2.138).

### 4.4. Analysis of Conserved Motifs and Gene Structures of Tomato SlCys Family Members

The sequences of tomato SlCys family proteins were submitted to MEME (https://meme-suite.org/meme/, accessed on 2 November 2024) to identify conserved motifs, and the maximum number of motifs was set to ten. Coding sequences (CDSs) and the untranslated region (UTRs) of tomato *SlCys* family genes were extracted according to the tomato genome annotation file. The conserved motifs and gene structures of tomato *SlCys* family members were visualized using TBtools (v2.138).

### 4.5. Phylogenetic Analysis of Cys Family Members

Cys family proteins in Arabidopsis and potato were identified as described above. Cys proteins of tomato, Arabidopsis and potato were submitted to the “One Step Build a ML Tree” plugin in TBtools (v2.138) to construct the phylogenetic tree of Cys family proteins (maximum likelihood method), with the bootstrap number set to 5000. Phylogenetic trees of tomato, Arabidopsis, and potato Cys family proteins were visualized using the iTOL website (https://itol.embl.de/, accessed on 3 November 2024).

### 4.6. Collinearity Analysis of Cys Family Genes

The tomato genome file and genome annotation file were submitted to TBtools (v2.138) to analyze the collinearity between *SlCys* genes in tomato. The genome files and genome annotation files of Arabidopsis and potato were downloaded from the Ensembl Plants database. The Arabidopsis and potato genome files, as well as the genome annotation files, were submitted together with the tomato genome file to TBtools (v2.138) to analyze the synteny between tomato *SlCys* genes and Arabidopsis and potato *Cys* genes.

### 4.7. Analysis of Promoter Cis-Acting Elements of Cys Family Genes in Tomato

The 2000 bp sequences upstream of the initial codon of the tomato *Cys* genes (promoter region) were extracted from the tomato genome file. Promoter sequences were submitted to the PlantCARE website (https://bioinformatics.psb.ugent.be/webtools/plantcare/html/, accessed on 3 November 2024) to identify *cis*-acting elements in the *SlCys* family gene promoters. The promoter *cis*-acting elements of tomato *Cys* family genes were visualized using TBtools (v2.138).

### 4.8. Transcriptome Data and qRT-PCR Analysis of SlCys Genes

The transcriptome data of tomato after cold treatment were downloaded from the NCBI website (https://www.ncbi.nlm.nih.gov/, accessed on 3 November 2024) using the data numbers for GSE148887. The FPKM values of *SlCys* genes were extracted according to gene ID and visualized using TBtools (v2.138).

Total RNA from tomato leaves was extracted at 0 h, 1 h, 2 h, 4 h, 8 h, and 12 h after cold treatment using FreeZol Reagent (Vazyme Biotech Co., Nanjing, China). cDNA was obtained by reverse transcription reaction using HiScript III RT SuperMix for qPCR (+gDNA wiper) (Vazyme Biotech Co., Nanjing, China). The primers used for qRT-PCR were designed using the Primer-BLAST tool on the NCBI website. The primers are listed in Table S2. The qRT-PCR assay was performed using ChamQ Universal SYBR qPCR Master Mix (Vazyme Biotech Co., Nanjing, China). Reaction times and temperatures were 5 min at 95 °C, 40 cycles of 95 °C for 10 s, 20 s at 60 °C, and 20 s at 72 °C. Three machine and biological replicates were performed for each sample. The relative expression level of *SlCys* genes were calculated using 2^−∆∆Ct^ method [34], and the *Slβ-actin* gene was selected as a reference gene.

### 4.9. Virus-Induced Silencing of SlCys5

The CDS of *SlCys5* was submitted to the VIGS tool of the SGN website (https://solgenomics.net/, accessed on 3 November 2024), and a *SlCys5*-silencing target with a length of 300 bp was designed. Primers (Cys5V-F: agtggtctctgtccagtcctATGGCGGGGGAAAAGACT; Cys5V-R: ggtctcagcagaccacaagtCATCGTAATGATGAGTTTGTAGCCT) were designed according to the target site and the vector sequence, and PCR amplification was performed. After product purification, the target sequence was ligated into the pPNC-TRV2 vector using the Nimble Cloning method [35]. The pTRV1 vector, pTRV2-00 empty vector, and pTRV2-*SlCys5* recombinant vector were transferred into *Agrobacterium tumefaciens* (GV3101). The bacteria containing the above vectors were cultured in IM medium for 24 h and resuspended in MgCl_2_-IM medium. The bacterial solution containing pTRV1 was mixed with equal volumes of bacterial solutions containing pTRV2-00 and pTRV2-*SlCys5*, respectively, and injected from the abaxial side of the leaf into 20-day-old tomato seedlings using a needle-free syringe. The tomatoes injected with the bacterial solution were treated in the dark for 24 h and then transferred to a plant incubator in a normal environment for cultivation. After 20 days, the expression of *SlCys5* was detected using the qRT-PCR method.

### 4.10. Physiological Assays

The contents of GSH and H_2_O_2_ in tomato leaves were measured using the commercial kits GSH-2-W and H_2_O_2_-1-Y (Suzhou Keming Biotechnology Co., Ltd., Suzhou, China). The rate of O_2_^−•^ production was detected according to the method proposed by Elstner and Heupel [36].

### 4.11. Statistical Analyses

The experimental data were statistically analyzed using SPSS (version 22) software, and the significance was analyzed using one-way ANOVA and two-way ANOVA.

## 5. Conclusions

In this study, we identified the *Cys* family at a genome-wide scale in tomato. We also detailed the chromosomal localization, protein motifs, gene structures, phylogeny, collinearity, *cis*-acting elements, and expression patterns of *SlCys* family members under cold stress. In addition, we verified the role played by *SlCys5* in cold tolerance in tomato. In summary, we elucidated the evolutionary pattern and functional differentiation of the *Cys* gene family in tomato, deepening our understanding of the regulatory mechanisms involved in cold stress tolerance in plants.

## Data Availability

The datasets used and/or analyzed during the current study are available from the corresponding author on reasonable request. However, most of the data are shown in the Supplementary Materials.

## Supplementary Materials

The supporting information can be downloaded at: https://www.mdpi.com/article/10.3390/ijms26062801/s1.

## References

[1] Birke H., Haas F.H., De Kok L.J., Balk J., Wirtz M., Hell R. (2012). Cysteine biosynthesis, in concert with a novel mechanism, contributes to sulfide detoxification in mitochondria of Arabidopsis thaliana. Biochem. J..

[2] Khare R., Kumar S., Shukla T., Ranjan A., Trivedi P.K. (2017). Differential sulphur assimilation mechanism regulates response of Arabidopsis thaliana natural variation towards arsenic stress under limiting sulphur condition. J. Hazard. Mater..

[3] Hell R., Bork C., Bogdanova N., Frolov I., Hauschild R. (1994). Isolation and characterization of two cDNAs encoding for compartment specific isoforms of O-acetylserine (thiol) lyase from *Arabidopsis thaliana*. FEBS Lett..

[4] Ikegami F., Itagaki S., Murakoshi I. (1992). Purification and characterization of two forms of cysteine synthase from Allium tuberosum. Phytochemistry.

[5] Koprivova A., Kopriva S. (2016). Hormonal control of sulfate uptake and assimilation. Plant Mol. Biol..

[6] Cui W., Chen H., Zhu K., Jin Q., Xie Y., Cui J., Xia Y., Zhang J., Shen W. (2014). Cadmium-induced hydrogen sulfide synthesis is involved in cadmium tolerance in Medicago sativa by reestablishment of reduced (homo) glutathione and reactive oxygen species homeostases. PLoS ONE.

[7] Cui W., Li L., Gao Z., Wu H., Xie Y., Shen W. (2012). Haem oxygenase-1 is involved in salicylic acid-induced alleviation of oxidative stress due to cadmium stress in Medicago sativa. J. Exp. Bot..

[8] Kawashima C.G., Noji M., Nakamura M., Ogra Y., Suzuki K.T., Saito K. (2004). Heavy metal tolerance of transgenic tobacco plants over-expressing cysteine synthase. Biotechnol. Lett..

[9] Dominguez-Solis J.R., Gutiérrez-Alcalá G., Romero L.C., Gotor C. (2001). The cytosolic O-acetylserine (thiol) lyase gene is regulated by heavy metals and can function in cadmium tolerance. J. Biol. Chem..

[10] Ning H., Zhang C., Yao Y., Yu D. (2010). Overexpression of a soybean O-acetylserine (thiol) lyase-encoding gene GmOASTL4 in tobacco increases cysteine levels and enhances tolerance to cadmium stress. Biotechnol. Lett..

[11] Alvarez C., Calo L., Romero L.C., GarcilA I., Gotor C. (2010). An O-acetylserine (thiol) lyase homolog with L-cysteine desulfhydrase activity regulates cysteine homeostasis in Arabidopsis. Plant Physiol..

[12] Bermudez M.A., Galmés J., Moreno I., Mullineaux P.M., Gotor C., Romero L.C. (2012). Photosynthetic adaptation to length of day is dependent on S-sulfocysteine synthase activity in the thylakoid lumen. Plant Physiol..

[13] Yamaguchi Y., Nakamura T., Kusano T., Sano H. (2000). Three Arabidopsis genes encoding proteins with differential activities for cysteine synthase and beta-cyanoalanine synthase. Plant Cell Physiol..

[14] Yuan Y., Song T., Yu J., Zhang W., Hou X., Ling Z.K., Cui G. (2021). Genome-wide investigation of the cysteine synthase gene family shows that overexpression of CSase confers alkali tolerance to alfalfa (*Medicago sativa* L.). Front. Plant Sci..

[15] Akbudak M.A., Filiz E., Uylas S. (2019). Identification of O-acetylserine (thiol) lyase (*OASTL*) genes in sorghum (*Sorghum bicolor*) and gene expression analysis under cadmium stress. Mol. Biol. Rep..

[16] Liu D., Li J., Lu J., Tian B., Liu X., Yang G., Pei Y. (2019). Cloning and functional analysis of four O-Acetylserine (thiol) lyase family genes from foxtail millet. Plant Physiol. Biochem..

[17] Carolina C., Francisco A.C. (2005). Tomato abiotic stress enhanced tolerance by trehalose biosynthesis. Plant Sci..

[18] Goulas E., Schubert M., Kieselbach T., Kleczkowski L.A., Gardeström P., Schröder W., Hurry V. (2006). The chloroplast lumen and stromal proteomes of Arabidopsis thaliana show differential sensitivity to short-and long-term exposure to low temperature. Plant J..

[19] Stitt M., Hurry V. (2002). A plant for all seasons: Alterations in photosynthetic carbon metabolism during cold acclimation in Arabidopsis. Curr. Opin. Plant Biol..

[20] Strand A., Hurry V., Henkes S., Huner N., Gustafsson P., Gardestrom P., Stitt M. (1999). Acclimation of Arabidopsis leaves developing at low temperatures. Increasing cytoplasmic volume accompanies increased activities of enzymes in the Calvin cycle and in the sucrose-biosynthesis pathway. Plant Physiol..

[21] Wang Y., Jiang H., Mao Z., Liu W., Jiang S., Xu H., Su M., Zhang J., Wang N., Zhang Z. (2021). Ethylene increases the cold tolerance of apple via the MdERF1B-MdCIbHLH1 regulatory module. Plant J..

[22] Nolan T.M., Vukašinović N., Liu D., Russinova E., Yin Y. (2020). Brassinosteroids: Multidimensional regulators of plant growth, development, and stress responses. Plant Cell.

[23] Meng F., Feng N., Zheng D., Liu M., Zhou H., Zhang R., Huang X., Huang A. (2024). Exogenous Hemin enhances the antioxidant defense system of rice by regulating the AsA-GSH cycle under NaCl stress. PeerJ.

[24] Van Hoewyk D., Pilon M., Pilon-Smits E.A.H. (2007). The functions of NifS-like proteins in plant sulfur and selenium metabolism. Plant Sci..

[25] Wang C., Zheng L., Tang Z., Sun S., Ma J.F., Huang X.-Y., Zhao F.-J. (2020). OASTL-A1 functions as a cytosolic cysteine synthase and affects arsenic tolerance in rice. J. Exp. Bot..

[26] Choi Y.E., Kwon K.W., Lee J.C., Woo S.Y. (2007). Expression of the rice cytoplasmic cysteine synthase gene in tobacco reduces ozone-induced damage. Plant Biotechnol. Rep..

[27] Li Z., Yang X., Li Z., Zou X., Jiang C., Zhu J., Zhang Y., Han Y., Liu C., Hao J. (2025). Genome-wide analysis of ABF gene family in lettuce (*Lactuca sativa* L.) reveals the negative roles of LsABF1 in thermally induced bolting. J. Hortic. Sci. Biotechnol..

[28] Zhou J., Hu F., Berhe M., Zhou R., Li D., Li H., Yang L., Zhou T., Zhang Y., Wang L. (2024). Genome-wide identification, classification, and expression profiling of LAC gene family in sesame. BMC Plant Biol..

[29] Baloch A.A., Kakar K.U., Rais S., Nawaz Z., Almoneafy A.A., Raza A.M., Khan S., Ullah R. (2025). Genome-wide analysis of CNGC gene family in *Brassica juncea* (L.) Czern reveals key targets for stress resistance and crop improvement. Plant Gene.

[30] Lian C., Li Q., Yao K., Zhang Y., Meng S., Yin W., Xia X. (2018). *Populus trichocarpa PtNF-YA9*, a multifunctional transcription factor, regulates seed germination, abiotic stress, plant growth and development in *Arabidopsis*. Front. Plant Sci..

[31] Hasanuzzaman M., Raihan R.H., Masud A.A.C., Rahman K., Nowroz F., Rahman M., Nahar K., Fujita M. (2021). Regulation of reactive oxygen species and antioxidant defense in plants under salinity. Int. J. Mol. Sci..

[32] Hasanuzzaman M., Bhuyan M.B., Zulfiqar F., Raza A., Mohsin S.M., Al Mahmud J., Fujita M., Fotopoulos V. (2020). Reactive oxygen species and antioxidant defense in plants under abiotic stress: Revisiting the crucial role of a universal defense regulator. Antioxidants.

[33] Chen C., Wu Y., Li J., Wang X., Zeng Z., Xu J., Liu Y., Feng J., Chen H., He Y. (2023). TBtools-II: A “one for all, all for one” bioinformatics platform for biological big-data mining. Mol. Plant.

[34] Livak K.J., Schmittgen T.D. (2001). Analysis of relative gene expression data using real-time quantitative PCR and the 2(-Delta Delta C(T)) method. Methods.

[35] Yan P., Zeng Y., Shen W., Tuo D., Li X., Zhou P. (2019). Nimble cloning: A simple, versatile, and efficient system for standardized molecular cloning. Front. Bioeng. Biotechnol..

[36] Elstner E.F., Adelheid H. (1976). Inhibition of nitrite formation from hydroxylammoniumch loride: A simple assay for superoxide dismutase. Anal. Biochem..

