# Supplementary material for: Identification of Cysteine synthase (Cys) Gene Family in Tomato (Solanum lycopersicum) and Functional of SlCys5 in Cold Stress Tolerance
Source: Int J Mol Sci. 2025 Mar 20;26(6):2801. doi: 10.3390/ijms26062801 (PMC11942816; doi:10.3390/ijms26062801)
Supplement: Supplementary file 1 [file ijms-26-02801-s001.zip › Table S1.pdf]

Table S1 *SlCys* family genes and physicochemical properties of SlCys proteins

Gene Name	Gene ID	Chr	aalen	MolWt	pI
SlCys1	Solyc01g094790.3.1	Chr1	357	38888.9	7.74
SlCys2	Solyc01g097950.3.1	Chr1	323	34369.81	5.28
SlCys3	Solyc07g065470.3.1	Chr7	421	45024.1	7.6
SlCys4	Solyc08g014340.3.1	Chr8	386	40939.18	5.41
SlCys5	Solyc09g082060.3.1	Chr9	325	34242.55	5.93
SlCys6	Solyc10g012370.3.1	Chr10	351	38237.11	8.39
