# Supplementary material for: Identification of Cysteine synthase (Cys) Gene Family in Tomato (Solanum lycopersicum) and Functional of SlCys5 in Cold Stress Tolerance
Source: Int J Mol Sci. 2025 Mar 20;26(6):2801. doi: 10.3390/ijms26062801 (PMC11942816; doi:10.3390/ijms26062801)
Supplement: Supplementary file 1 [file ijms-26-02801-s001.zip › Table S2.pdf]

Table S2 Primers for qRT-PCR experiments

Primer	Sequence
qCys1F	GGCTACAAAATGGTTTTGACTATGCCAT
qCys1R	TGTCGATTCCAAAAGATCATAAGCCT
qCys2F	CATTATGAAGCAACTGGACCTGAAATAT
qCys2R	TTTCTGGATTCTTTTCCTTGAGAAACTT
qCys3F	AAAAACTCAAATATCAAGTGCTTCCTTA
qCys3R	TATTCCAATCCCTTCGGTTATTGTAT
qCys4F	CCCTTAACTTCCCTCTGTAACACT
qCys4R	GGCACAGATACTGCTTTGCAAAC
qCys5F	GAGAGAAGAATTATTCTGCGTGCTT
qCys5R	TTAGCAGGGTTTTCAAATTGCTGAAGA
qCys6F	GTTGGTGAGGAGAAGATTTTATTCGAG
qCys6R	ACAGCTCGAAGCACTGAGAATATGAA
qACTIN-F	ACAACTTTCCAACAAGGGAAGAT
qACTIN-R	TGTATGTTGCTATTCAGGCTGTG
